# The crucial factor of hospital readmissions: a retrospective cohort study of patients evaluated in the emergency department and admitted to the department of medicine of a general hospital in Italy

**DOI:** 10.1186/s40001-014-0081-5

**Published:** 2015-01-27

**Authors:** Fabio Fabbian, Arrigo Boccafogli, Alfredo De Giorgi, Marco Pala, Raffaella Salmi, Roberto Melandri, Massimo Gallerani, Andrea Gardini, Gabriele Rinaldi, Roberto Manfredini

**Affiliations:** Clinica Medica, Department of Medical Science, University of Ferrara, 44124, Cona Ferrara, Italy; Department of Medicine, Azienda Ospedaliera-Universitaria (AOU) of Ferrara, 44124, Cona Ferrara, Italy; 2nd Unit of Internal Medicine, Department of Medicine, AOU of Ferrara, Ferrara, Italy; Emergency Department, AOU of Ferrara, Ferrara, Italy; 1st Unit of Internal Medicine, Department of Medicine, AOU of Ferrara, Ferrara, Italy; Medical Director, AOU of Ferrara, Ferrara, Italy; General Manager, AOU of Ferrara, Ferrara, Italy

**Keywords:** Hospitalization, Readmission, Length of stay, Mortality, Comorbidity, Internal medicine

## Abstract

**Background:**

Early hospital readmissions, defined as rehospitalization within 30 days from a previous discharge, represent an economic and social burden for public health management. As data about early readmission in Italy are scarce, we aimed to relate the phenomenon of 30-day readmission to factors identified at the time of emergency department (ED) visits in subjects admitted to medical wards of a general hospital in Italy.

**Methods:**

We performed a retrospective 30-month observational study, evaluating all patients admitted to the Department of Medicine of the Hospital of Ferrara, Italy. Our study compared early and late readmission: patients were evaluated on the basis of the ED admission diagnosis and classified differently on the basis of a concordant or discordant readmission diagnosis in respect to the diagnosis of a first hospitalization.

**Results:**

Out of 13,237 patients admitted during the study period, 3,631 (27.4%) were readmitted; of those, 656 were 30-day rehospitalizations (5% of total admissions). Early rehospitalization occurred 12 days (median) later than previous discharge. The most frequent causes of rehospitalization were cardiovascular disease (CVD) in 29.3% and pulmonary disease (PD) in 29.7% of cases. Patients admitted with the same diagnosis were younger, had lower length of stay (LOS) and higher prevalence of CVD, PD and cancer. Age, CVD and PD were independently associated with 30-day readmission with concordant diagnosis and kidney disease with 30-day rehospitalization with a discordant diagnosis.

**Conclusions:**

Comorbid patients are at higher risk for 30-day readmission. Reduction of LOS, especially in elderly subjects, could increase early rehospitalization rates.

## Background

Early hospital readmissions, defined as rehospitalization within 30 days from a previous discharge, represent an important economic and social burden for public health management, especially at a time with a widespread and severe paucity of economic resources. Moreover, rehospitalizations are considered ‘sentinel events’, and, in October 2012, the Federal Government of the USA activated a control program of hospital readmission, penalizing the hospitals with higher readmissions rates [1,2]. In our country, several regional laws have introduced economic penalties in the case of readmissions as well. In our region (Emilia-Romagna), a recognized top-level health-care region in Italy, since 1999 all hospital readmissions within 30 days with same discharge diagnosis receive an economic penalty consisting of 20%-50% of their reimbursement or even of 100% in the case of readmission within 0–1 days [3]. Therefore, if the discharge diagnosis after readmission is the same as that of the previous discharge, a penalty will be applied; on the contrary, it will not be applied if the discharge diagnosis after readmission is different. Analysis of US data shows that large hospitals and teaching hospitals are the most penalized [4], although the reason is not clear. Health administrations would like to improve patients’ care quality and reduce costs at the same time [5], so careful reduction of the length as well as cost of the hospital stay is considered an efficacious strategy. Thus, assessment of early hospital readmissions represents a parameter for measuring the quality of treatment, although the problem is still under investigation and needs to be better understood. Several studies have evaluated the proportion of readmissions in different hospital settings, and for departments of internal medicine, the average of readmission rate was between 12 and 27% of total hospitalizations [6,7]. The principal causes of hospitalizations are chronic diseases, including heart failure and chronic lung disease, i.e., chronic obstructive pulmonary disease (COPD) and asthma [8,9]. On the one hand, evaluation of readmissions is gaining importance worldwide because of the demographic changes and the greater demand for hospital beds. On the other hand, data about rehospitalization in Italian hospitals are scarce.

The aim of this study was to relate the phenomenon of 30-day readmission to factors identified at the time of ED visits in subjects hospitalized in a department of medicine in a general hospital in Italy.

## Methods

This observational, cross-sectional study, conducted with the approval of the local institutional committee for human research between January 2010 and July 2012, evaluated all patients admitted to the Department of Medicine of St. Anna Hospital, Ferrara, Italy. St. Anna Hospital is a 600-bed teaching hospital with almost all facilities, except for cardiothoracic surgery. It serves as the main hospital of the city of Ferrara (≈150,000 inhabitants) and as a hub center for the entire province of Ferrara (other 210,000), which has two other smaller second-level hospitals. The annual flow of patients in the ED is approximately 76,000. The province of Ferrara is characterized by a high percentage of elderly subjects (26% are older than 65 years), and approximately 3,000 subjects are aged ≥ 90 years. We analyzed the age, sex and reason for hospitalization of all patients admitted to the Department of Medicine. The Department of Medicine consists of four Internal Medicine units, two Infectious Disease units, and one each of Geriatrics and Gastroenterology (165 total beds, open for ED admissions for 24/24 h and 7/7 days). About one third of all hospital admissions are directed to the Department of Medicine. Most of the medical and nursing staff is permanent, also covering holidays. Length-of-hospital stay (LOS) and in-hospital mortality (IHM) were also calculated. Medical diseases leading to hospitalization were arbitrarily defined by classifying disease symptoms into subgroups: hematologic/oncologic, cardiovascular, pulmonary, neurologic, renal and gastrointestinal diseases. Moreover, the presence of a positive history of surgery was also considered, whereas musculoskeletal, cutaneous and other diseases were classified as miscellaneous. Oncologic disease included the presence of a malignancy in every organ. The details of the considered diseases are reported in Table 1. We performed a retrospective 30-month observational study focusing on two groups of subjects, those readmitted within 30-month and 30-day time periods. Patients with a rehospitalization within 30 days were evaluated on the basis of the admission diagnosis, and rehospitalization was considered as a concordant diagnosis if the ED diagnosis was the same as for the previous ED presentation; otherwise, readmission was considered discordant. Therefore, the primary classification was based on the second admission diagnosis: concordant or discordant in respect to the previous ED diagnosis. Factors associated with diagnosis of early readmission were also evaluated. The 30-month time period (i.e. 2,5 years) was arbitrarily chosen for comparison with the 30-day time period.

**Table 1:** **Medical diseases leading to hospitalization**

Cardiovascular disease	Heart failure, infectious, inflammatory, ischemic, valvular diseases, and venous or arterial system diseases
Pulmonary disease	Infectious, inflammatory and vascular damage of lungs or pleura
Neurologic disease	Degenerative and ischemic damage of the central nervous system
Renal disease	All processes responsible of acute or chronic reduction of renal function
Gastrointestinal disease	Hepatic damage with liver dysfunction, biliary damage due to infectious, inflammatory or dysplastic processes with and without jaundice and gallstones, acute and chronic pancreatitis, infectious, inflammatory or dysplastic processes of stomach and bowel, including diverticulitis
Hematologic disease	All processes leading to alterations of blood cells, excluding post-hemorrhagic anemia
Post-surgery condition	All patients who underwent any recent operation on any organ
Musculoskeletal disease	All processes leading to altered function of bone and muscles
Cutaneous disease	All processes altering the skin

Results are shown as mean ± SD or percentage. Clinical parameters were compared by the *t*-test, chi-squared and Mann–Whitney *U* test as appropriate. Logistic regression analysis was conducted in order to evaluate the characteristics related to readmission within the 30-day period. Age (as a continuous variable), sex, LOS and medical diseases leading to hospitalization were the independent variables. Moreover, the same analysis was carried out in order to investigate the relationship between the same independent variables and 30-day readmission with a concordant or discordant ED diagnosis, the latter being the dependent variable. Statistical analysis was performed using the SPSS 13.0 software (SPSS, version 13, SPSS Inc., Chicago, IL, USA).

## Results and discussion

During the 30-month study period, 13,237 consecutive patients were admitted to the Department of Medicine (mean age 76.6 ± 13.5 years; 43.4% males), and 3,631 patients (27.4%, 1-year readmitted patients 1,452) were readmitted. Six hundred fifty-six subjects (5% of total admissions) were readmitted within 30 days (mean 13.3 ± 8.5 days; median 12 days) from the previous discharge. In this latter group of patients, the diagnosis was not different (i.e., was concordant) from the previous one in 316 (48.1%) cases. Causes of 30-day readmissions were cardiovascular disease in 29.3% of patients, pulmonary disease in 29.7% and other reasons in 28.4% (Table 2). Data regarding the main characteristics of readmissions within the 30-month period were not different (data not shown). Univariate analysis did not show a difference between readmissions within the 30-day and 30-month periods (n = 2,975), in sex distribution or LOS; on the other hand, the 30-day readmitted subjects were younger than the 30-month ones (78.2 ± 11.3 vs. 79.8 ± 10.7 years; *p* < 0.001) (Table 3).

**Table 2:** **Main characteristics and reasons for readmission within the 30-day time period (** ***n*** **= 656)**

Age (years ± SD)	78.2 ± 11.3
Males (*n*, %)	272 (41.5%)
Females (*n*, %)	384 (58.5%)
Cardiovascular disease	192 (29.3%)
Pulmonary disease	195 (29.7%)
Gastrointestinal disease	110 (16.8%)
Neoplastic disease	82 (12.5%)
Neurological disease	66 (10.1%)
Hematological disease	21 (3.2%)
Surgery disease	3 (0.5%)
Kidney disease	44 (6.7%)
Miscellaneous	186 (28.4%)
Length-of-hospital stay (days)	8.4 ± 6.8
Days prior to admission (days)	13.3 ± 8.5

**Table 3:** **Univariate analysis comparing readmission within 30 days and 30 months**

	**30-day readmission (** ***n*** **= 656)**	**30-month readmission (** ***n*** **= 2975)**	***p***
Age (years)	78.2 ± 11.3	79.8 ± 10.7	<0.001
Males (%)	24.4%	76.6%	NS
Females (%)	24.9%	75.1%	
LOS first admission (days)	9.2 ± 7.4	8.9 ± 7.6	NS
LOS readmission (days)	8.6 ± 7	8.6 ± 7.2	NS

Evaluation of patients with an ED diagnosis concordant and discordant with the previous hospitalization (Table 4) showed that the former group was younger (76 ± 13 vs. 81 ± 8 years, *p* < 0.001) and had a lower LOS during the previous hospitalization (7.9 ± 6.9 vs. 8.9 ± 6.8 days, *p* = 0.01) and a higher prevalence of readmission because of cardiovascular (54.7% vs. 45.3%, *p* = 0.032), pulmonary (56.4% vs. 43.6%, *p* = 0.006) and cancer (60.5% vs. 32.5%, *p* = 0.018) diseases. On the contrary, readmissions because of neurological diseases (36.4% vs. 63.6%. *p* = 0.043), renal diseases (27.3% vs. 72.7%, *p* = 0.004) and miscellaneous diseases (38.7% vs. 61.3%, *p* = 0.002) were more frequent in patients admitted with a discordant ED diagnosis.

**Table 4:** **Univariate analysis comparing patients readmitted within the 30-day time period with concordant and discordant diagnoses**

	**Concordant diagnosis ** **(** ***n*** **= 316)**	**Discordant diagnosis (** ***n*** **= 340)**	***p***
Age (years)	76 ± 13	81 ± 8	<0.001
Sex (male/female)	140/176	132/208	NS
Deceased (*n* = 115)	52 (45.2%)	63 (54.8%)	NS
Admission due to cardiovascular disease (*n* = 192)	105 (54.7%)	87 (45.3%)	0.032
Admission due to pulmonary disease (*n* = 195)	110 (56.4%)	85 (43.6%)	0.006
Admission due to neurological disease (*n* = 66)	24 (36.4%)	42 (63.6%)	0.043
Admission due to gastrointestinal disease (*n* = 110)	46 (41.8%)	64 (58.2%)	NS
Admission due to neoplastic disease (*n* = 82)	49 (60.5%)	32 (39.5%)	0.018
Admission due to renal disease (*n* = 44)	12 (27.3%)	32 (72.7%)	0.004
Miscellaneous (n = 186)	72 (38.7%)	114 (61.3%)	0.002
Total time in the ED	2:05 ± 2:58	2:08 ± 1:26	0.015
Length of stay, previous admission (days)	7.9 ± 6.9	8.9 ± 6.8	0.010
Days prior to admission (days)	13.5 ± 8.3	13.2 ± 8.7	NS

Age [OR = 1.014 (95% CI: 1.007-1.020), *p* < 0.001] and LOS of the first hospitalization [OR = 0.903 (95% CI: 0.819-0.996), *p* = 0.041] were independently associated with the risk of 30-day rehospitalization. Age [OR = 1.055 (95% CI: 1.037-1.073), *p* < 0.001], readmissions due to cardiovascular disease [OR = 1.819 (95% CI: 1.261-2.622), *p* = 0.001] and pulmonary disease [OR = 1.759 (95% CI: 1.223-2.53), *p* = 0.002] increased the risk of 30-day rehospitalization with a concordant ED diagnosis, while readmissions due to kidney disease were independently associated with 30-day rehospitalization with a discordant ED diagnosis [OR = 0.445 (95% CI: 0.219-0.905), *p* = 0.025] (Table 5).

**Table 5:** **Logistic regression analysis evaluating factors independently associated with readmissions within the 30-day time period and the 30-day time period with a concordant diagnosis**

**Factors related to rehospitalization within 30 days**	**OR**	**95% ** **CI**	***p***
Age (continuous variable)	1.014	1.007 − 1.020	<0.001
LOS of previous hospitalization	0.903	0.819 − 0.996	0.041
**Factors related to rehospitalization within 30 days with a concordant diagnosis**	**OR**	**95% ** **CI**	***p***
Age (continuous variable)	1.055	1.037 − 1.073	<0.001
Admission due to cardiovascular disease	1.807	1.234 − 2.646	0.002
Admission due to pulmonary disease	1.814	1.229 − 2.679	0.003
Admission due to kidney disease	0.449	0.220 − 0.915	0.027

In-hospital mortality of 30-day readmitted patients was 17.5%, which was higher than the in-hospital mortality of readmitted patients within the 30-month period (10.1%) (*p* < 0.0001).

In this study, conducted in an Italian internal medical department, readmissions within 30 months and 30 day were 27.4% and 5%, respectively. Thirty-day readmitted patients were slightly younger than 30-month ones, and age and shorter LOS of the previous hospitalization increased the risk of early secondary hospitalization. Thirty-day readmitted patients with a concordant diagnosis with respect to the previous one (mainly classified as cardiovascular or pulmonary disease) were younger and had a lower LOS of the previous hospitalization than 30-day rehospitalized subjects with a discordant diagnosis. The 30-day rehospitalization rate was relatively low, but the analysis was limited to a department of medicine only.

The problem of 30-day readmissions has been known for a long time, and it has been related to the quality of care. In the mid 1990s, early rehospitalization rates varying from 5% to 29% were reported, which is not so different from our findings [10]. Data from the available studies are summarized in Table 6. In their prospective study conducted on patients discharged from three large acute hospitals in California, Florida and Nebraska, Vashi *et al.* calculated that for every 1,000 discharges, there were 97.5 ED treat-and-release visits and 147.6 hospital readmissions in the 30 days following discharge [7]. Moreover, a retrospective cohort study conducted on administrative data from an US urban academic center demonstrated that out of 15,519 patients who had been previously discharged, 3,695 (23.8%) had at least one visit in the ED within the subsequent 30 days [11]. In the present study, we did not evaluate all the ED visits, but only those followed by hospital admission, so that probably the ED reevaluation rate would had been much higher than 5 and 27.4%. Dharmarajan *et al.* evaluated Medicare free-for-service beneficiaries and found that the 30-day readmission rate was 24.8% for heart failure, 19.9% for myocardial infarction and 18.3% for pneumonia [12]. The proportion of patients readmitted for the same condition was 35.2%, 10%, and 22.4%, respectively, and median time to 30-day readmission was between 10 and 12 days [13]. Recently, Donzé *et al.* [9] reported that the prevalence of 30-day readmission between 2009 and 2010 was around 22%, and infection, cancer, heart failure, gastrointestinal and liver disorders were the most frequent primary diagnoses of potentially avoidable readmissions. On the other hand, data from a systematic review [14] did not identify any precise risk factor that could predict patients’ readmission.

**Table 6:** **Hospital readmissions: available data in the literature**

**Author**	**Study period**	**Country**	**Type of study**	**Patients (** ***n*** **)**	**Readmission rate**
Vashi *et al.* [7]*.*	Jul 2008 − Sep 2009	USA	Prospective	4.028,555	14.7%
Rising *et al.* [11]	Jan − Jun 2010	USA	Retrospective	15,519	23.8%
Dharmarajan *et al.* [12]	2007 − 2009	USA	Retrospective	3.047,615	21.0%
Donzè *et al.* [9]	2009 − 2010	USA	Retrospective	10,731	22.3%
Moloney *et al.* [15]	2002	Ireland	Retrospective	4,051	15.2%
Kaboli *et al.* [16]	1997 − 2010	USA	Observational	4.124,907	15.7%
Dobrzanska *et al.* [17]	Sep 2002 − Aug 2003	UK	Observational	1,235	11.6%
Maurer *et al.* [18]	Mar − May 1998	France	Retrospective	773	12.3%
Jencks *et al.* [19]	2003 − 2004	USA	Retrospective	11.855,702	19.6%
Hasan *et al.* [20]	Jul 2001 − Jun 2003	USA	Prospective observational	10,946	17.5%
Glynn *et al.* [21]	2002 − 2008	Ireland	Retrospective	23,114	27.0%

In agreement with other studies [15], although the mean age of our population was rather high, we found that age was associated with rehospitalization. We also found that a lower LOS was associated with early rehospitalization, a relationship that has been reported since 1986 [22] and further confirmed by other authors [10,15]. In contrast, a large study on about 4 million medical hospital admissions [16] showed that a reduction in the LOS was not associated with a worsening of rehospitalization, but each additional day over the average of LOS was associated with a relative increase of 3% in the probability of readmission. The latter study was the only one showing that a more rapid hospital discharge did not expose to increased risk of rehospitalization, rather a reduction of 27% of the average LOS [−1.46 days (5.44 to 3.98)] was associated with a reduction in 16% of early rehospitalization [16]. The study conducted by Jencks *et al.* [19] on more than 13 million patients in the US showed that 30-day rehospitalizations were ascribed more frequently to medical than surgical departments (21.1% vs. 15.6%). The highest rehospitalization rates were identified for heart failure (26.9%), pulmonary diseases, including COPD (22.6%) and pneumonia (20.1%), psychoses (24.6%) and gastrointestinal disorders (19.2%). In this study, the risk of hospitalization increased with age, male gender and the presence of end-stage renal disease. Bisharat *et al.* [6] also reported the importance of renal disease in the readmission rate. Our study showed that patients with renal disease (probably as well as those with neurologic disease) were readmitted with different diagnoses from the previous one, suggesting a higher rate of drug side effects in older people with decreased renal function [23].

As a confirmation of the role of age and comorbidity in causing hospital readmission, a relationship between mortality and early readmission has been shown, with in-hospital mortality being higher in readmitted subjects [21]. Comorbidity was also the cause of early readmission in other smaller Italian studies [24,25].

Our study has several limitations. First, the study design is retrospective and evaluates patients admitted to a single hub center, which is probably not representative of the reality in community hospitals. Second, the Italian health care organization endorses different regional health authorities to organize outpatient follow-up of chronic conditions in a different way. Ferrara is a town located close to the boundaries of two other regions with different health-care organizations, and our hospital may also admit subjects living in these areas. Third, we did not classify readmissions on the basis of validated algorithms, which could have identified potentially avoidable readmissions, although the readmission rate was low. Fourth, reasons for readmission were arbitrarily classified, even if the diagnosis was made by experienced physicians. On the other hand, functional status, therapy and social support were not analyzed. Fifth, we did not consider any vital clinical parameters for the analysis, such as blood pressure, heart rate, breathing rate or oxygenation. However, it should be underlined that, due to the low rate of early readmission, probably only subjects with alteration in vital clinical parameters were admitted. Last but not least, although it has been shown that preventable readmissions were mainly due to clinician-related and patient-related factors, and the risk for avoidable readmissions included longer LOS, higher number of hospitalizations and attendance in public outpatient clinics and EDs in the past year [26], in our study we did not take these factors into account. In fact, our aim was merely to analyze the presence of any relationship between ED evaluation and readmissions. In the previously quoted study, Yam *et al.* [26] concluded that, when limiting the analysis to unplanned readmissions, only the concordance of the principal diagnosis and shorter period of time before readmission were associated with avoidable rehospitalizations. Anyway, the present study also has the strength of having considered more than 13,000 patients over a 2.5-year period.

## Conclusions

Early hospital readmissions are considered an indicator of quality of care and represent a global concern in terms of patient safety, adverse drug events, health-care associated infections, procedural complications, disease or functional decline exacerbations, and also economic costs. The present study shows that age remains a risk factor for early rehospitalization of elderly patients. A significant reduction of the number of available hospital beds has been the consequence of a rationalization of all public expenses, including those for health assistance (free for all citizens in Italy). This reduction has led to the need for a stricter and more appropriate utilization of hospital beds and control of readmissions. Thus, after discharge from acute hospital settings, specific territorial network paths, based on specific patient needs, should be strongly recommended.

## Abbreviations

ED: Emergency department
CVD: Cardiovascular disease
PD: Pulmonary disease
LOS: Length of stay
COPD: Chronic obstructive pulmonary disease
IHM: In-hospital mortality
